# Evaluating the impact of an interdisciplinary integrated limb preservation service operating concurrently with a single‐specialty service

**DOI:** 10.1002/jfa2.12013

**Published:** 2024-04-13

**Authors:** Sebouh Bazikian, Alyssa J. Pyun, Hanke Zheng, William Padula, Tanzim Khan, Kenneth Ziegler, Laura Shin, Gregory A. Magee, Vincent L. Rowe, David G. Armstrong

**Affiliations:** ^1^ Keck School of Medicine University of Southern California Los Angeles California USA; ^2^ Division of Vascular Surgery and Endovascular Therapy Department of Surgery Keck School of Medicine University of Southern California Los Angeles California USA; ^3^ Department of Pharmaceutical and Health Economics Alfred E. Mann School of Pharmacy and Pharmaceutical Sciences University of Southern California Los Angeles California USA; ^4^ Leonard D. Schaeffer Center for Health Policy and Economics University of Southern California Los Angeles California USA; ^5^ Division of Vascular Surgery and Endovascular Therapy David Geffen School of Medicine University of California Los Angeles California USA

**Keywords:** diabetic foot ulcers, limb preservation, lower extremity amputation, podiatry, vascular surgery

## Abstract

**Background:**

This study examined the efficacy of an interdisciplinary limb preservation service (LPS) in improving surgical outcomes for diabetic foot ulcer (DFU) patients compared to traditional care.

**Methods:**

Data from January 1, 2017 to September 30, 2020 were retrospectively reviewed. An interdisciplinary LPS clinic began on August 1, 2018, coexisting with a preexisting single specialty service. Primary outcomes were major/minor amputation rates and ratios and hospital length of stay. Surgical endpoints pre‐ and post‐LPS launch were compared.

**Results:**

Among 976 procedures for 731 unique DFU patients, most were male (80.4%) and Hispanic (89.3%). Patient demographics were consistent before and after LPS initiation. Major amputation rates decreased by 45.5% (15.4%–8.4%, *p* = 0.001), with outpatient procedures increasing over 5‐fold (3.3% pre‐LPS to 18.7% post‐LPS, *p* < 0.001). Hospital stay reduced from 10.1 to 8.5 days post‐LPS (*p* < 0.001). The major to minor amputation ratio declined from 22.4% to 12.7%.

**Conclusions:**

The interdisciplinary LPS improved patient outcomes, marked by fewer major amputations and reduced hospital stays, suggesting the model's potential for broader application.

## 1 BACKGROUND

Lower extremity complications of diabetes inflict a significant personal and financial burden. They frequently result in personal distress, depression, and a heavy financial burden on the healthcare system [1, 2]. A significant proportion of the annual $176 billion expenditure for diabetes care in the US is associated with lower‐extremity complications, primarily driven by risk factors such as diabetic neuropathy and peripheral artery disease (PAD) [3, 4]. Despite the crucial need for timely treatment, considerable barriers persist in diabetic foot ulcer (DFU) management, particularly, the observed disparities in amputation rates among minority groups [5]. Data strongly suggest that a comprehensive, coordinated, interdisciplinary approach may help address these systemic challenges, including fragmented care, treatment delays, and lack of coordination—all of which contribute to these disparities and adverse outcomes [6, 7, 8, 9].

While there is a preexisting Single‐Specialty Foot Service (SSS) that has been running for more than 4 decades at one of our key teaching hospitals, we established a service with an interdisciplinary Limb Preservation Service (LPS) in August 2018 in parallel to improve patient care. This created an opportunity to study the impact of the interdisciplinary service on limb preservation as the patient population remained similar before and after the implementation of LPS.

The LPS establishes a central contact point for coordinating multifaceted care for limb‐threatening conditions related to diabetes, ischemia, or infection [10]. This approach streamlines decision‐making for referring physicians, effectively managing lower‐extremity complications with a single call, reducing hospital stay length, and shifting surgical focus from reactive and ablative procedures to proactive and reconstructive ones. The "hot foot line" is activated based on rapid evaluation of a patient's condition, typically upon identification of tissue loss, signs of infection or ischemia, or suspicion of osteomyelitis. Subsequent triage involves consultation with the appropriate specialist, such as a podiatrist or vascular surgeon, with additional collaboration as needed from other specialties such as vascular medicine or endocrinology to maximize positive outcomes.

Therefore, this study aims to evaluate the impact of the LPS on surgical outcomes in patients with complex, limb‐threatening DFUs in a center with a long‐standing single‐specialty service.

## 2 METHODS

We abstracted data from January 1, 2017, to September 30, 2020, based on the natural experiment of implementing the LPS on August 1, 2018. We compared data from the preexisting SSS from January 2017 to August 2018 and data post‐August 2018 from the ongoing SSS and the newly established LPS. The study protocol was approved by the Institutional Review Board (IRB).

### 2.1 Data source and study population

We reviewed the data from the charts of adult patients who received surgeries for diabetic foot complications at two clinics of a county hospital system, including the preexisting SSS and the LPS. The surgeries of interest included any surgery with the preoperative diagnosis including DFUs and its associated complications. Subsequent revisions related to the index operation were also included.

The SSS evaluated was managed by an orthopedic surgeon. Following the institution of the LPS, both shared the response to DFU consultations within the system equally based on clinical and surgical availability. As both the SSS and LPS were designed to treat people with diabetes, we excluded the surgery records of patients who did not have diabetes and those who underwent traumatic amputations.

### 2.2 Explanatory variables

The key explanatory variable was the implementation of the interdisciplinary LPS. Patient demographic and clinical characteristics were also abstracted from each surgical record, including age, gender, ethnicity, comorbidities, and smoking status.

### 2.3 Endpoints

The primary endpoints included major amputation proximal to the foot (Hi), minor amputation (Lo), Hi/Lo ratio, and hospital length of stay (LOS). Major amputation was defined as any amputations at or above the ankle, whereas minor amputation referred to amputations below the ankle. In addition, we evaluated the use of noninvasive diagnostic imaging, vascular consult, vascular interventions, and outpatient procedures.

### 2.4 Statistical analysis

Patient characteristics were described based on the information at the first encounter. Using procedure level data, we evaluated the level of LPS by comparing the outcomes before and after its implementation. The data were structured longitudinally by patient‐time (pre‐ or post‐LPS). Procedures were organized into the pre‐LPS group if the surgery was performed in the SSS before August 2018; all other procedures conducted post‐August 2018 were categorized into the post‐LPS group. For patients with multiple procedures on record, they were clustered into categories: ipsilateral minor/major amputation, debridement or other procedure (tendon balancing, etc.), and contra‐lateral minor/major amputation, debridement, or other procedures. We reported the unadjusted statistics of study endpoints pre‐ and post‐launch of the LPS and by the two clinics. Student's *t*‐test and/or Analysis of variance (ANOVA) test were applied to assess the difference in numeric variables, and the chi‐square test was performed to compare the difference in categorical variables.

We performed a multivariable logistic regression to assess the degree of association between LPS or SSS on the probability of major amputation, controlling for patient demographic and clinical features. We also performed generalized linear regression with a log link and gamma distribution to evaluate the impact of these settings on hospital LOS. Data for these regression models were structured longitudinally by patient‐time, accounting for the pre–post period when LPS was introduced. We tested our longitudinal data model for random effects (a random intercept) to capture between‐patient variability in baseline risk of amputation. Using the Hausman test, the fixed effects form of the model was rejected in favor of a random intercept. A statistically significant level of 0.05 for a two‐sided *p*‐value was applied.

## 3 RESULTS

A total of 976 surgical procedure records of 731 unique patients were identified and included in this analysis. Overall, the mean (SD) age of these patients at their first encounter was 54 (9.4) years and the majority were male (80.4%) and Hispanic (89.3%) patients. The patient demographic and medical history was similar before and after the initiation of LPS program, indicating a comparable demographic and medical history profile in both phases of the study. These data are further illustrated in Table 1. Surgical procedures were categorized by the implementation of the LPS–390 procedures conducted pre‐launch (in the preexisting SSS) and 586 taking place post‐launch of the interdisciplinary LPS (414 in the preexisting SSS and 172 in the new LPS). In addition, 202 (27.6%) patients had more than one procedure either on the index extremity or contralateral extremity.

**TABLE 1:** Patient characteristics.

Characteristic	Overall		Pre‐LPS phase (SSS clinic only)		Post‐LPS phase (SSS + LPS clinic)		*p* value
	*N*	%	*N*	%	*N*	%	
Age							
Mean (SD)	54.0 (9.4)		53.8 (9.3)		54.2 (9.4)		0.6
≤50	259	35.4%	105	34.4%	154	36.2%	0.45
50–60	278	38.0%	124	40.7%	154	36.2%	
≥60	194	26.5%	76	24.9%	118	27.7%	
Male	588	80.4%	241	79.0%	347	81.5%	0.41
Ethnicity		0.0%		0.0%		0.0%	
White	37	5.1%	12	3.9%	25	5.9%	0.35
Hispanic	653	89.3%	279	91.5%	374	87.8%	
Black	27	3.7%	11	3.6%	16	3.8%	
Asian	12	1.6%	3	1.0%	9	2.1%	
Other	2	0.3%	0	0.0%	2	0.5%	
Comorbidities		0.0%		0.0%		0.0%	
Hypertension	536	73.3%	228	74.8%	308	72.3%	0.4
Hyperlipidemia	329	45.0%	134	43.9%	195	45.8%	0.09
CAD	63	8.6%	25	8.2%	38	8.9%	0.27
CKD	123	16.8%	48	15.7%	75	17.6%	0.66
ESRD	57	7.8%	25	8.2%	32	7.5%	0.81
Smoking	250	34.2%	120	39.3%	130	30.5%	0.62

Abbreviations: CAD, coronary artery disease; CKD, Chronic Kidney Disease; ESRD, End‐Stage Renal Disease; LPS, limb preservation service; SD, standard deviation; SSS, single‐specialty foot clinic.

### 3.1 Amputation and relevant surgical outcomes

**FIGURE 1:**
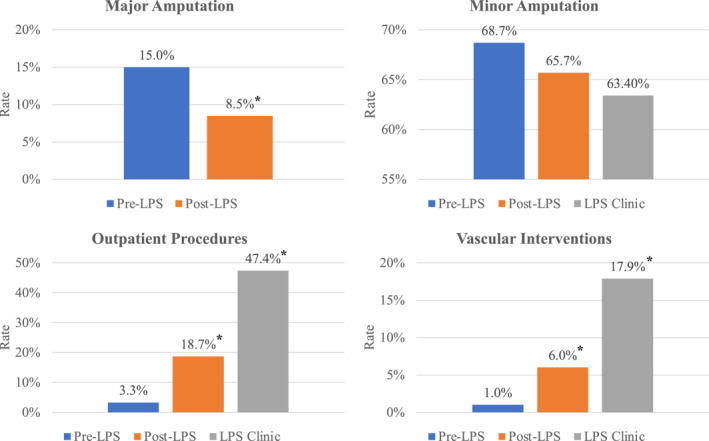
Effects of implementation of interdisciplinary LPS. The percentage was calculated based on the number of total procedures performed in the period/setting specified. Pre‐LPS refers to the period in which only the SSS operated. Post‐LPS refers to the period in which both the LPS and SSS operated. LPS, limb preservation service; SSS, single specialty service. * indicated the *p* < 0.001.

The rate of major amputation significantly decreased from 15.4% to 8.4% (*p* < 0.001), leading to a substantial reduction in the Hi/Lo amputation ratio from 22.4% to 12.7% (Figure 1). A numeric decrease in the rate of limb‐sparing minor amputation was observed after the launch of the LPS (68.7% pre‐LPS vs. 65.7% post‐LPS, *p = *0.33). There was more than 5‐fold increase in outpatient procedures (3.3% pre‐LPS vs. 18.7% post‐LPS, *p < *0.001*),* and the increase was attributable to the interdisciplinary LPS clinic, achieving 47.4% in outpatient procedures. These outpatient procedures included debridement, tendon balancing procedures, and limb‐sparing minor amputations. The rate of vascular interventions increased 6‐fold from 1.0% to 6.0% (*p < *0.001) in the post‐launch, which was mostly due to the interdisciplinary LPS clinic which integrated a vascular intervention in 17.9% of cases.

There were 42 revascularizations related to the index DFU (4 in pre‐LPS and 38 post‐LPS). Of these interventions, endovascular procedures accounted for 69% (29), surgical revascularization represented 24% (10), and hybrid interventions constituted 7% (3). Furthermore, 20 (48%) were associated with minor amputations, 2 (5%) were associated with major amputations, and the rest, 20 (47%), were associated with debridement or other procedures. Among 38 patients with at least 1‐year follow‐up, 1(2.6%) patient died and 4 (10.3%) had a major amputation. In addition, there was a significantly higher vascular consult rate in the interdisciplinary LPS clinic compared to the SSS post‐launch (13.1% in LPS pre‐launch period, 15.5% in LPS post‐launch period, 33.7% in the LPS clinic, *p < *0.001). Of note, major amputations are noted as 0 in the LPS clinic because those procedures are beyond the scope of services offered by the clinic and were redirected to the SSS. These data are further illustrated in Table 2.

**TABLE 2:** Comparison of surgical outcomes.

	Pre‐LPS phase (SSS clinic only)		Post‐LPS phase (SSS + LPS clinic)		LPS clinic only		*p* value
	*N*	%	*N*	%	*N*	%	
Any amputation	328	84.1%	434	74.1%	109	63.4%	**<0.001**
Major amputation	60	15.4%	49	8.4%	0	0.0%	**<0.001**
Minor amputation	268	68.7%	385	65.7%	109	63.4%	0.33
Hi/Lo ratio		22.4%		12.7%		N/A	
Noninvasive diagnostic imaging	100	25.6%	154	26.3%	46	26.7%	0.96
Vascular consult	51	13.1%	91	15.5%	58	33.7%	0.29
Vascular intervention	4	1.00%	38	6.00%	34	17.9%	**<0.001**
Outpatient procedure	14	3.30%	119	18.7%	90	47.4%	**<0.001**
Mean LOS (SD), day	10.1 (7)		8.5 (7.6)		7.2 (4.9)		**<0.001**

*Note*: *p* values were generated from the comparison between hospital outcomes between pre‐ and post‐launch of the LPS. The percentage was calculated based on the number of total procedures performed in the period/setting specified.

Abbreviations: Hi/Lo, major/minor amputation rate; LOS, length of hospital stay; LPS, limb preservation service; SD, standard deviation; SSS, single‐specialty foot clinic.

The results of multivariate logistic regression are presented in Table 3. Adjusting for patient characteristics, the implementation of the LPS was associated with a 50% reduction in the likelihood of experiencing a major amputation (Odds Ratio [OR] = 0.5, *p* < 0.001).

**TABLE 3:** Adjusted analysis of risk of major amputation.

Parameters	OR	95% CI	*p* value
Interdisciplinary LPS	0.5	[0.33, 0.75]	**<0.001**
Age			
≤50	Reference		
50–60	1.43	[0.89, 2.32]	0.14
>60	1.17	[0.68, 2.01]	0.56
Male	1.64	[0.93, 2.90]	0.09
Hispanic	0.76	[0.40, 1.43]	0.39
Comorbidities			
Hypertension	1.61	[0.93, 2.77]	0.09
Hyperlipidemia	1.06	[0.69, 1.62]	0.79
CAD	1.62	[0.83, 3.12]	0.15
CKD	1.17	[0.70, 1.97]	0.55
ESRD	0.59	[0.24, 1.45]	0.25
Smoking	1.18	[0.78, 1.80]	0.43
Intercept	0.08	[0.03, 0.21]	**<0.001**

Abbreviations: CAD, coronary artery disease; CI, Confidence Interval; CKD, Chronic Kidney Disease; ESRD, End‐Stage Renal Disease; LPS, Limb Preservation Service; OR, odds ratio.

### 3.2 Length of hospital stay

For the procedures performed in the inpatient setting, the average (SD) LOS decreased from 10.1(7) to 8.5 (7.6) days after the implementation of the LPS (*p = *0.001). The decrease was largely driven by the LPS with the average LOS of 7.2 (4.9) days. According to the multivariate generalized linear regression, the implementation of the LPS was significantly associated with a shorter LOS (*p = *0.004).

## 4 DISCUSSION

Our study underlines the substantial influence of a LPS on DFUs management. We found a significant decrease in major amputation rates from 15.4% to 8.4%, following LPS implementation. No substantial changes were observed in minor amputations. Moreover, this led to a marked reduction in the Hi/Lo ratio from 22.4% to 12.7%. A multivariate logistic regression analysis revealed that the LPS was linked with a 50% decrease in the risk of major amputation. Post‐LPS, there was a significant increase in outpatient procedures (3.3%–18.7%) and vascular interventions (1.0%–6.0%). For inpatient procedures, we noted an average LOS decrease from 10.1 to 8.5 days post‐LPS.

One novel aspect of this study was that it compared service outcomes with those of a prior SSS that maintained operation throughout the study period. Moreover, the substantial sample size and high percentage of Hispanic patients bring new dimensions to the study and provides valuable insights for future planning and development of programs aimed at similar patient populations.

Reinforcing prior research and systemic reviews, we emphasized the vital role of interdisciplinary teams in enhancing limb preservation for DFU patients [9, 11]. Specifically, our findings are supported by evidence from 18 studies involving 38,608 participants, which demonstrated a significant reduction in major amputations following the implementation of an interdisciplinary care team, with a pooled OR of 0.5, similar to our reported OR of 0.48 [1, 12] The encouraging reduction in major amputation and Hi/Lo ratio post‐LPS implementation further highlights the efficacy of this approach. Given the high morbidity and mortality associated with major amputations in DFU patients, our findings highlight the crucial role of an LPS [13, 14].

Furthermore, Hispanic populations often face increased rates of diabetes, DFUs, and major amputations [5, 15, 16]. Indeed, data from our team and others suggest that Hispanic individuals with diabetes‐related foot ulcers have at least a 33% higher risk of major amputation and a 10% higher risk of minor amputation than White individuals [13]. Implementing the LPS in a population with a high percentage of Hispanic patients has the potential to begin to address these disparities. While we did not identify glaring differences between Hispanic and non‐Hispanic patients with the numbers available, we strongly believe that this is a question worth addressing across an entire health system. In fact, the provisional success of the LPS has led our county to develop what may be the first interdisciplinary county‐wide limb preservation working group in the USA [17].

The implementation of the program led to a 15% reduction in LOS, translating into considerable cost savings. An LPS in New Mexico led to the annual cost savings of approximately $1.4 million due to a decreased hospital LOS. The estimated cost savings, considering the procedural and facility costs of amputations along with hospital LOS, increased to approximately $2.9 million post‐implementation [18]. This has been corroborated by other studies [19, 20]. Moreover, the rate of procedures performed in an outpatient setting rose by 450% after the implementation of the LPS. We report a similar reduction in LOS even while keeping the SSS in the pool of patients. We believe this may be due to several factors, including paying more attention to discharging patients to home rather than keeping patients in for repeat inpatient procedures.

Our study has several limitations. The study was conducted at a single institution, which may limit the generalizability of the findings to other settings. The population studied was quite homogenous, predominantly male, and Hispanic, which may not reflect the diversity of patients with DFUs in other regions or countries. Another limitation is the lack of use of billing codes, which could have provided a more accurate comparison of procedures between the SSS and LPS. Additionally, this study did not evaluate other important nonoperative interventions of the services involved, including wound care and medical management. Furthermore, we were not yet able to capture even longer‐term outcomes, including mortality and revision procedures. Finally, while the patient characteristics and comorbidities of both groups studied appeared balanced (Table 1), we were unable to reliably apply a Wound, Ischemia, foot Infection score to the SSS. Efforts are underway to address the above limitations as well as changes and potential savings in utilization by the units themselves as well as emergency department and other downstream services.

In conclusion, our study underscores the considerable influence of an LPS on the management of DFUs. With a significant reduction in major amputations, among other benefits, the advantages of implementing an LPS are evident. Importantly, our study reveals that an LPS can coexist and function effectively alongside a preexisting SSS without requiring the dissolution or replacement of the SSS. Rather than being mutually exclusive, the LPS and SSS can coexist and supplement one another. For example, the LPS redirected major amputations to the SSS, while the LPS emphasized more complex and interdisciplinary limb salvage solutions. The value of an interdisciplinary approach, as evidenced by the LPS model, cannot be overstated. Our study amplifies the need for varied healthcare specialists to collaborate, thereby maximizing the potential for optimal patient outcomes [1, 9]. This study advocates for the adoption of similar interdisciplinary strategies in other healthcare settings, aiming to enhance patient outcomes and improve the quality of life for those with DFUs.

## 5 CONCLUSION

Our research highlights the significant impact of an interdisciplinary LPS in managing DFUs. The implementation of LPS resulted in a notable reduction in major amputations and other significant benefits, even when operating alongside the SSS. The findings underscore the fact that both services can complement each other effectively and operate concurrently. The interdisciplinary nature of the LPS model emphasizes collaboration among diverse healthcare specialists, leading to optimal patient outcomes. Given these benefits, our findings advocate for broader adoption of interdisciplinary approaches, such as the LPS, in other healthcare contexts to improve outcomes and quality of life for DFU patients.

## AUTHOR CONTRIBUTIONS

**David G. Armstrong**: Conceptualization; writing – original draft; writing – review & editing; formal analysis. **Sebouh Bazikian**: Data curation; writing – original draft; writing – review & editing; conceptualization. **Alyssa J. Pyun**: Data curation; writing – review & editing; conceptualization. **Hanke Zheng**: Conceptualization; writing – original draft; writing – review & editing; formal analysis. **William Padula**: Conceptualization; writing – review & editing; formal analysis. **Tanzim Khan**: Writing – review & editing; conceptualization. **Vincent L. Rowe**: Writing – review & editing; conceptualization.**Gregory A. Magee**: Writing – review & editing; conceptualization. **Kenneth Ziegler**: Writing – review & editing; conceptualization. **Laura Shin**: Writing – review & editing; conceptualization.

## CONFLICT OF INTEREST STATEMENT

HZ reports receiving a research stipend from Bristol Myers Squibb irrelevant to the submitted work.

## ETHICS STATEMENT

This study was approved by the Rancho Research Institute under the IRB approval number #460 on November 16, 2020.

## CONSENT FOR PUBLICATION

Not applicable as no identifying personal information is being published in this manuscript.

## Data Availability

Data is available upon reasonable request.
